# Recent advances in understanding the role of proteostasis

**DOI:** 10.12703/r/10-72

**Published:** 2021-09-15

**Authors:** Kanika Verma, Monika Verma, Aseem Chaphalkar, Kausik Chakraborty

**Affiliations:** 1 CSIR-Institute of Genomics and Integrative Biology, Mathura Road, Delhi, India; 2 Academy of Scientific and Innovative Research, CSIR-HRDC, Ghaziabad, Uttar Pradesh, India

**Keywords:** Proteostasis, protein folding, protein degradation, molecular chaperones, Unfolded Protein Response, aging, metabolism, autophagy, neurodegeneration

## Abstract

Maintenance of a functional proteome is achieved through the mechanism of proteostasis that involves precise coordination between molecular machineries assisting a protein from its conception to demise. Although each organelle within a cell has its own set of proteostasis machinery, inter-organellar communication and cell non-autonomous signaling bring forth the multidimensional nature of the proteostasis network. Exposure to extrinsic and intrinsic stressors can challenge the proteostasis network, leading to the accumulation of aberrant proteins or a decline in the proteostasis components, as seen during aging and in several diseases. Here, we summarize recent advances in understanding the role of proteostasis and its regulation in aging and disease, including monogenetic and infectious diseases. We highlight some of the emerging as well as unresolved questions in proteostasis that need to be addressed to overcome pathologies associated with damaged proteins and to promote healthy aging.

## Defining the need to understand the role of proteostasis

Proteins need to be maintained in their native structures at required cellular concentrations and specific locations. Deviations lead to aberrant enzyme activity, binding, stoichiometry in complexes, or aggregation, all of which may impose a penalty on cellular fitness, finally leading to ailing pathophysiological states or cell death. Proteostasis is the homeostasis of maintaining the proteome by regulating protein synthesis, translocation (if required), post-translational modifications (if required for stabilizing folded states), folding, and degradation or conversion of toxic oligomers into benign and less toxic amyloid aggregates^1^. An effective interplay between components of the proteostasis network consisting of chaperones (protein and chemical), co-chaperones, degradation machinery, translation control machinery, and adaptor proteins take cares of the balance in proteostasis. Hence, a thorough understanding of proteostasis will allow us to not only tackle the protein folding problems at hand but also open gates to a healthy aging paradigm.

## Proteostasis components, problems, and regulation

### Components of the proteostasis network

**Figure 1.**
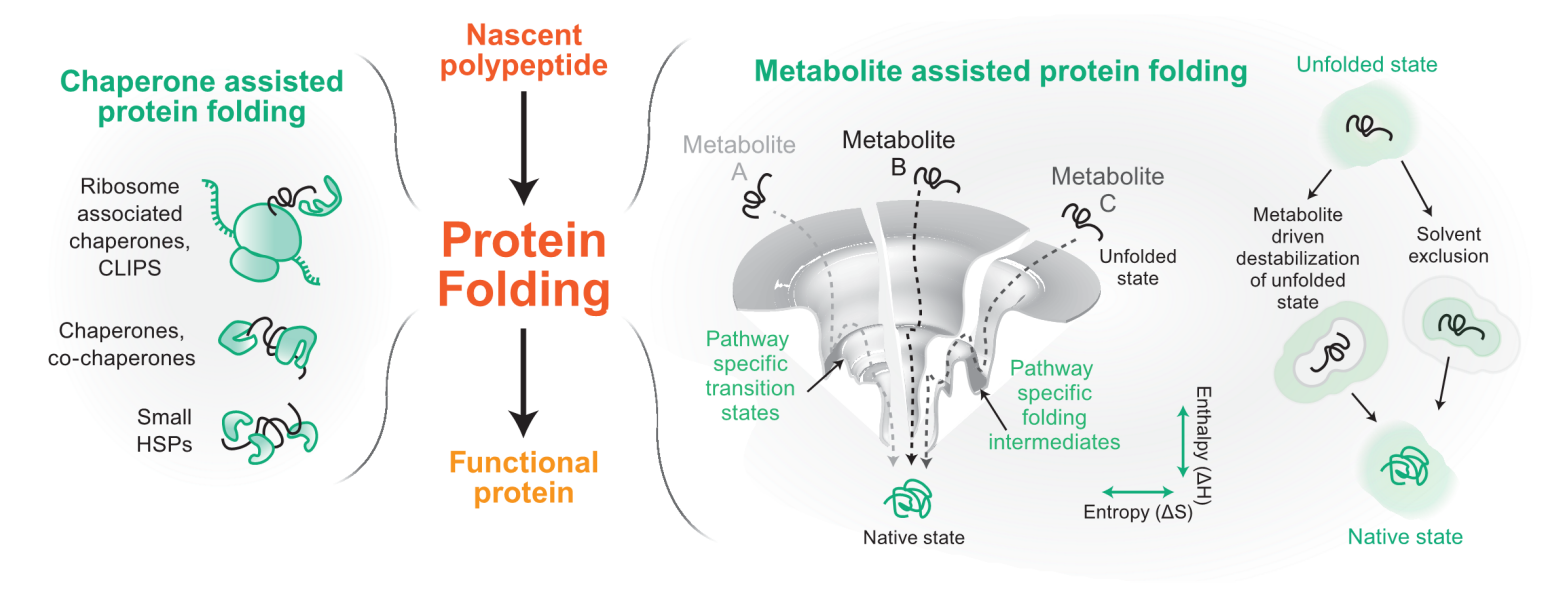
Components of proteostasis helping in protein folding. From its conception on the ribosome, a protein molecule folds to its native functional state with the help of two major proteostasis components. The left panel shows some of the representative components of a chaperone-based protein folding arm. In the right panel, we show metabolite-assisted folding of a protein. The folding landscape of a protein has been shown in the presence of three metabolites A, B, and C. On the extreme right, possible molecular mechanisms of metabolite-based protein folding are depicted. Gray represents solvent and green represents metabolite. CLIPS, chaperones linked to protein synthesis; HSP, heat shock protein.

Proteins are translated on ribosomes that also act as scaffolds to assemble protein chaperones, which aid the nascent chains to reach their final functional structure. Protein chaperones recognize and bind nascent chains or folding intermediates that exhibit exposed hydrophobic patches^2^. Binding and unbinding of the substrate protein with its chaperone follow a cycle dictated by either the ATP-hydrolysis rate of the chaperone/co-chaperone system (like Hsp70, Hsp90, and Hsp60 chaperone systems)^3,4^ or the dissociation and association rate of the substrate on ATP-independent chaperones (small heat shock proteins)^5^ (Figure 1). Ribosome-associated chaperones are called the CLIPS (chaperones linked to protein synthesis), which act as guardians of the nascent chains and comprise the Hsp70, Hsp40, Hsp110, and Hsp60 group of chaperones^6^. However, the Hsp70 system can perform diverse functions, including disaggregation of amyloid fibrils owing to its binding to a myriad of Hsp40s and Hsp110s as co-chaperones^7–9^. After protein translation, translocation is mediated by cytosolic and compartment-specific chaperones. Many of the proteins destined for cytosol, nucleus, and peroxisomes are folded in the cytosol by a cycle of binding and unbinding to the protein chaperones^10^. For proteins destined to the endoplasmic reticulum (ER) and mitochondria, the nascent chains are maintained in an unfolded state at the site of translation, targeted to the appropriate compartment, and subsequently folded inside either post-translationally or co-translationally using the chaperone machinery present in the targeted organelle^11^.

Each organelle that handles non-native protein chains has its complement of protein chaperones to aid folding. ER, which handles most of the membrane and secretory proteome, contains specialized chaperones that help in N-glycosylation and disulphide bond formation along with ER-localized forms of the canonical Hsp70 and Hsp90 chaperone machineries^12^. The mitochondrial matrix (MM), on the other hand, handles a large number of metabolic enzymes and proteins involved in oxidative phosphorylation; it has chaperones that are homologous to the Hsp70, Hsp90, and Hsp60 class of chaperone systems. Mitochondrial intermembrane space (IMS) resembles ER in terms of oxidative folding and has disulphide-forming chaperones along with other small chaperones. This space is thought to be devoid of the canonical Hsp70, Hsp90, and Hsp60 chaperoning machineries^13^.

Protein chaperones have diverged and specialized for organelle-specific proteostasis. Although the protein chaperones in the different compartments play a central role in protein quality assurance, recent reports have shown that the cellular milieu comprising the different metabolites also plays an important role in assisting proteins to their native states^14–17^. The exact mechanism of metabolite assistance is unknown, but metabolites may change the solvent structure or interaction of the nascent chain with the solvent or also aid the process of folding directly or indirectly through protein chaperones^18–20^ (Figure 1). More importantly, these reports have opened new avenues by placing metabolites into the proteostasis network.

### Problems in proteostasis

A hitch in any of the steps of proteostasis can lead to its disequilibrium. It may start when one or more proteins take more than their allocated time to reach its folded state. Although this timer mechanism is important to ensure that the proteins are given sufficient time to fold to their native structure before tagging it as a non-foldable protein, evidence for such a mechanism is prominently established only in the ER^21^. A protein that has a problem in folding can be degraded either by the collaboration of chaperones, C-terminal Hsp70-interacting protein (CHIP), BAG3, ubiquitin ligase, and proteasome or by the chaperone-dependent autophagy^22,23^. In some cases, when both are unable to take care of the misfolded protein, it may sequester essential chaperones and other proteins to form small soluble aggregates that confer cellular toxicity as seen for multiple neurodegenerative diseases^24^. Alternatively, the misfolded protein may be packaged into vesicles to form harmless molecular aggregates^25,26^. These small aggregates can also be detoxified by cellular processes that coagulate them to form larger seemingly harmless aggregates^27^.

**Figure 2.**
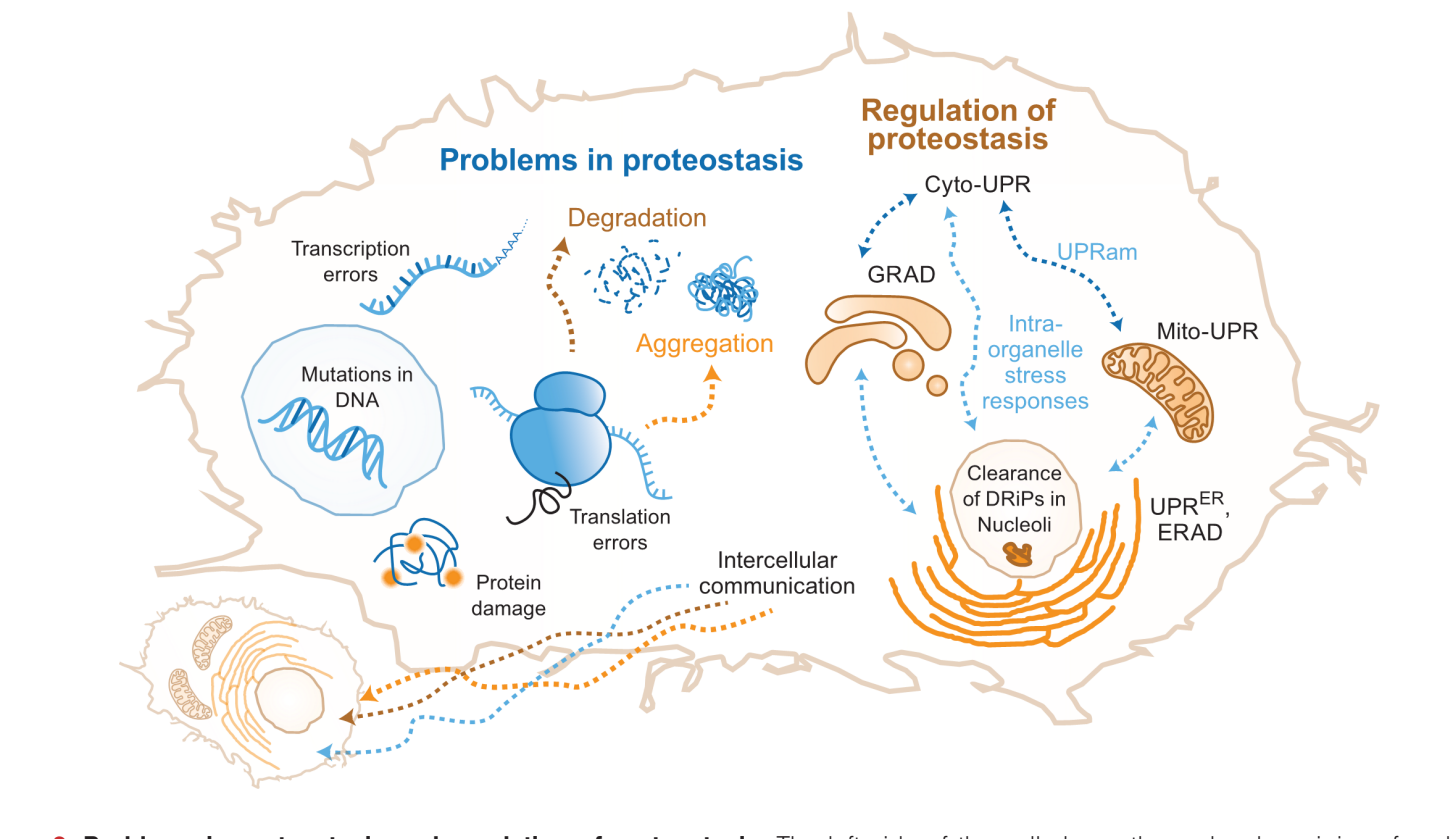
Problems in proteostasis and regulation of proteostasis. The left side of the cell shows the molecular origins of problems in protein folding. Errors at various steps in the life cycle of a protein leading to protein degradation and protein aggregation, resulting in imbalance of proteostasis. On the right, a few of the different organelle-specific cellular responses to the imbalance of proteostasis are shown. They include inter-organelle and inter-cellular responses. Cyto-UPR, cytosolic unfolded protein response; DRiP, defective ribosomal product; ERAD, endoplasmic reticulum–associated degradation; GRAD, Golgi apparatus–related degradation; Mito-UPR, mitochondrial unfolded protein response; UPRam, unfolded protein response activated by mistargeting of proteins; UPR^ER^, endoplasmic reticulum–associated unfolded protein response.

Proteostasis problems can also start with an incompletely translated nascent chain resulting from a damaged mRNA or an mRNA with base misincorporation, faulty editing, or splicing (Figure 2). These nascent chains are actively degraded by the ribosome quality control (RQC) system that couples protein and damaged mRNA degradation^28,29^. Mistranslated proteins are ubiquitinated and transported to the nucleolus for degradation^30^. Mislocalization of the proteins can also precipitate problems; if ER proteins are mistargeted to mitochondria (or vice versa) during stress, incompatibility between the chaperone systems and the mistargeted proteins leads to the loss of proteostasis^31,32^.

Even folded proteins may initiate proteostasis disequilibrium due to environmental insults that result in protein damage and aggregation^33^ (Figure 2). These aggregates need to be disaggregated or cleared while damaged proteins need to be degraded to prevent aberrant interactions and signaling events.

### Regulation of proteostasis machinery

Problems in proteostasis are sensed in the different compartments by specialized sensors. Heat shock factor 1 (HSF1) is the canonical eukaryotic sensor in the cytosol for proteostasis disequilibrium and is maintained in an inactive state by the Hsp70 and Hsp90 chaperones. When misfolded proteins accumulate, they titrate out the chaperones and free up HSF1, which then is free to translocate to the nucleus to signal cytosolic unfolded protein response (cyto-UPR)^10,34^. HSF1 activation upregulates chaperones, degradation machinery, and metabolic and stress response–related genes. More recently, heme-regulated kinase (HRI) was discovered as an additional cytosolic sensor of protein aggregation arising because of blocked protein degradation, which attenuates protein translation to decrease the intracellular protein concentration^35^.

In the ER, proteostasis disequilibrium is sensed by IRE1 (the most conserved sensor among all eukaryotes), ATF6, and PERK (the last two branches are currently known to be present only in metazoans). These sense proteostasis problems in the ER to elicit an integrated stress response that increases ER chaperones, components of ER-associated degradation (ERAD) (clears misfolded proteins of ER) or autophagy, and ER volume (to decrease protein concentration)^36^. This concerted response decreases global protein translation and alters metabolism among a host of other pathways that are still being investigated^37^. Although IRE1 and ATF6 seem to be redundant in the pathways that they regulate, PERK primarily prevents protein translation by phosphorylating eIF2α and inactivating it. Recent studies of these signaling processes have shed light on the nuances that govern this complex integration of stress response^38,39^. Activation of the ER sensors also seems to follow the same titration model as HSF1, where the ER-resident Hsp70 (Bip/GRP78) prevents activation of the sensors as long as it is not titrated away by the accumulation of the client proteins^40^.

Whereas regulation of proteostasis machinery in the ER is well studied, little is known about proteostasis regulation in the mitochondria. Mitochondria, being a double-membrane-bound organelle, creates additional complexity with different compartments vis-à-vis MM and IMS having different redox environments. Proteostasis stress due to mitochondrial DNA damage is sensed by ATFS-1, first discovered in *Caenorhabditis elegans* to upregulate mitochondrial chaperones in response^41^. Subsequently, ATF5 was found to play a similar role in mammalian cells^42^. However, mitochondria respond differently to misfolding inside the MM (ROX1)^43^ and proteostasis imbalance due to overburdened protein translocation machinery^44^. The MM along with the nucleolus^45,46^ plays an important role in clearing cytosolic misfolded proteins. Nucleoli and promyelocytic leukemia protein bodies act as clearance hubs for defective ribosomal products as well^30^. Currently, there is limited knowledge regarding proteostasis disequilibrium in the two sub-compartments and whether they can report the compartment-specific problems back to the nucleus. Given the importance of mitochondria in global misfolding stress, sensors and signaling mechanisms of the two sub-compartments are of immediate interest. In a recent study, Rao *et al*. showed sub-compartment-specific stress-response pathways in MM and IMS during proteotoxic stress-induced by misfolded proteins, where upregulation of TOM complex mediates the IMS response while Vms1 is important in MM stress response^47^.

As stress may disturb organellar membranes, reorganization of some membrane-bound organelles is also reported during stress. For example, Golgi apparatus–related degradation is aided by 26S proteasome in the cytosol and controls Golgi dispersal^48^. Another study reported the shuttling of mammalian ubiquitin ligase CHIP from chaperones to the membranes during acute stress. CHIP then acts on its organelle specific substrates leading to reorganization of that organelle^49^. Remarkably, the stress-response pathways of the different compartments seem to communicate intracellularly: ER-UPR activation can clear cytoplasmic aggregates^50,51^, and downregulation of cytosolic chaperones can upregulate ER stress response in specific cells^52^ (Figure 2). Similarly, mitochondrial proteostasis can take care of cytosolic misfolded proteins^45^, whereas ER can act as a hub for protein quality control when mitochondrial proteostasis is perturbed^53^. Also, mitochondrial precursor proteins, when accumulated in the cytosol, result in the activation of another type of stress response, referred to as UPRam (UPR activated by mistargeting of proteins), which was found to be beneficial for cells^54^. Another study discovered similar mitochondrial precursor over-accumulation stress (mPOS) in *Saccharomyces cerevisiae* disturbing cytosolic proteostasis^55^. Response to mPOS includes upregulation of ribosome-associated proteins, ultimately leading to cell survival^55^. Thus, the stress-response pathways in organelles are interconnected and this connection extends to inter-organelle contact sites as well. For example, contact sites of mitochondria with lysosomes and ER are quite important for mitochondrial quality control (reviewed elsewhere^56^). Organisms not only have intracellular stress sensors and response mechanisms but the response pathways are also regulated by cell non-autonomous signaling^57^. Intercellular communication (Figure 2) of this sort is seen in *C. elegans*, where the upregulation of mitochondrial unfolded protein response (Mito-UPR) in the brain seems to activate the HSF1-dependent pathway in the intestinal cells^58,59^. ER-UPR upregulation in the glial cells of *C. elegans* leads to the upregulation of ER-UPR in distal cells using neuropeptide signaling conferring ER stress tolerance and longevity^60^. In a striking discovery, a tyrosine phosphatase that negatively regulates HSF1 activity was found to be downregulated by endogenous small interfering RNA (endo-siRNA) in germline-less *C. elegans*^61^. This suggests that in metazoans multiple pathways upregulate the proteostasis network even when intracellular signals are missing, thus opening new avenues to modulate proteostasis using hormonal or neurotransmitter-based signaling in organisms^62–64^.

Although we have a large catalogue of proteostasis members and pathways that regulate it, the knowledge is by no means exhaustive. New members of the proteostasis network are being identified not only in complex metazoans but also in simple and well-studied model organisms like *Escherichia coli* and *S. cerevisiae*^65,66^. There are many unknowns in the way that proteostasis network members are regulated. For example, we have only started to understand whether different types of misfolded proteins in the same compartment would signal similar or different pathways and how the amplitude and the spectrum of response would depend on the number of misfolded molecules and their type^67^. The regulation of these members is being studied, and quantitative models have been developed to simulate the proteostasis network of different organisms^68,69^. The ever-increasing knowledge in this field contributes immensely to our understanding of the role of proteostasis in health and diseases.

## Aging and proteostasis

**Figure 3.**
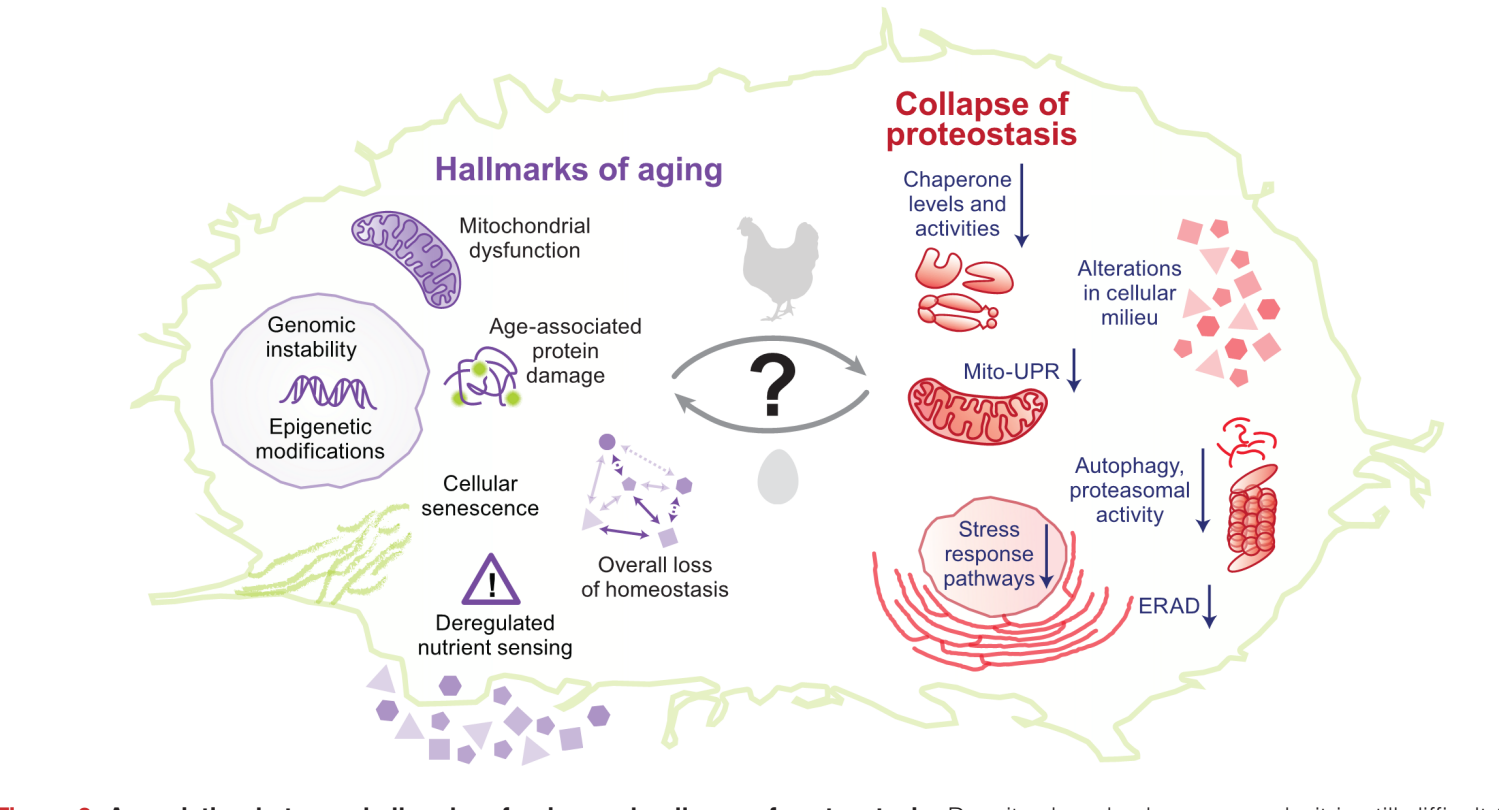
Association between hallmarks of aging and collapse of proteostasis. Despite decades-long research, it is still difficult to pinpoint to a definite cause-and-effect relationship between hallmarks of aging and collapse of proteostasis. On the left, a few important cellular hallmarks of aging are shown. On the right, age-associated global changes to proteostasis are shown. ERAD, endoplasmic reticulum–associated degradation; Mito-UPR, mitochondrial unfolded protein response.

Multiple pieces of evidence suggest that aging leads to a progressive decline in proteostasis^70–72^. Unstable transgenic proteins are found to form punctate structures in aged *C. elegans*, and a large fraction of the endogenous proteome aggregates in an age-dependent manner^73–75^. In an interesting short-lived vertebrate model, *Nothobranchius furzeri*, protein aggregates comprising primarily ribosomal subunits increased with age, indicating a loss of ribosome stoichiometry, aggregation, and proteostasis imbalance^76^. Another comprehensive proteomics study reported that mice showed only minimal change in proteome with age^77^; however, the authors focused only on the soluble proteome and its alteration with age. Hence, it is possible that the aggregated proteome changes with age. Direct correlation of protein aggregation with age was shown by an aggregated protein fraction from the brain of young mice having enhanced ability to seed Aβ aggregation in aged mice, underlining their compromised proteostasis^78^. Supporting this, in humans, amyloid-type aggregates show an age-dependent increase in plasma^79^. Environmental and genetic conditions that lead to delayed aging also delay age-associated protein aggregation in *C. elegans*^74^. However, the connection between aging and proteostasis is not simple; rather, aging in metazoans is a complex process involving multiple cell types, and each cell type may have a different rate of molecular aging determining their dependence on proteostasis^80^. Although aging leads to a decline in proteostasis, a decline in proteostasis may also contribute to aging: expression of aggregation-prone proteins in worms and mouse decreases their life span and accelerates aging-associated phenotypes^24,74^. Other conditions that perturb proteostasis, like the mismatch between mitochondrial and nuclear genotype, can also accelerate aging^81^. Evidence from the “anti-aging” program in stem cells also suggests that aging is partially governed by the maintenance of proteostasis^82–84^. Proteostasis decline makes up for the primary hallmarks of aging along with genomic instability, epigenetic modifications, and telomere attrition. Secondary hallmarks or antagonistic hallmarks—deregulated nutrient sensing, cellular senescence, and mitochondrial dysfunction—arise after that, leading to tertiary hallmarks which are seen as phenotypes when homeostasis is not restored^85^. Since these hallmarks can occur simultaneously and continuously during aging, the connection between aging and proteostasis is two-way: proteostasis decline accelerates aging while aging accelerates the decline of proteostasis (Figure 3).

The reasons behind the age-dependent decline of proteostasis are not clear since many factors can dictate proteostasis collapse: (1) accumulation of age-dependent damage to proteins, (2) deregulation of protein synthesis causing aberrant accumulation of proteins involved in functional complex formation^86^, (3) the inability of aged cells to respond to altered proteostasis demands^87,88^, and (4) alterations in cellular milieu because of age-dependent changes in metabolism and hence impairment of the metabolite-dependent folding arm of proteostasis^17,58,89^. Any of these factors either alone or in combination could lead to the age-dependent collapse of proteostasis. The major focus has been on the age-associated accumulation of damaged proteins and the age-dependent loss of responsivity of stress-response pathways that maintain proteostasis (Figure 3).

### Protein damage

Proteins accumulate damage during aging^90^. Modifications such as carbonylation, oxidation, glycation, and deamidation can change protein structure, function, and aggregation propensity of the folded proteins, thereby causing global protein aggregation^91,92^. Pathways nullifying this damage can delay aging, indicating that this damage may have a significant contribution in increasing the load on the proteostasis network during aging^93^. Interestingly, pathways that alleviate this damage are thought to play an important role in life-span extension when organisms are treated with sub-lethal stresses early in life (a phenomenon termed hormesis)^51^. Although damage to proteins may play a crucial role in determining the load on the proteostasis network, error in mRNA editing, mRNA splicing, and translation fidelity can also increase the load on the proteostasis network by producing faulty proteins that are unable to fold^94–96^.

Protein misfolding may escalate during aging if damaged proteins are not cleared efficiently. Supporting this, increasing protein degradation by enhancing autophagy^97^ or proteasomal pathway^98^ enhances life span^99^. Although chemically inducing autophagy by rapamycin increases life span^100^, it also represses protein translation, a well-known modifier of aging^101^. The specific role of autophagy was substantiated by upregulating the adaptor protein, p62, which increases autophagy and hence life span in both *C. elegans*^102^ and *Drosophila melanogaster*^103^. The link between life span and the capacity to clear the damaged proteins is further validated by the observations that long-lived mammals tend to have enhanced protein degradation pathways^104^. However, it is important to understand whether there is a threshold above which the increase of the degradation pathways, particularly autophagy, causes deleterious effects^105^. Given the benefit of upregulating these pathways and the threshold, these studies are really helpful for designing therapeutics wherever needed.

### Age-dependent loss of proteostasis network–restoring pathways

Some of the stress-response pathways that regulate proteostasis exhibit an abrupt programmed decrease with aging, specifically during the reproductive phase of worms, partially explaining the age-dependent loss of proteostasis^88,106,107^. Experiments with mammals have shown conflicting results including decrease or no change in heat shock response (HSR) with aging^108,109^. Even while this is being investigated, the consensus is that proteostasis-restoring pathways decline with age. For example, protein turnover through the autophagic flux marginally decreases with age^110^, and in a vertebrate model, killifish, a decrease in expression of proteasome components could predict the life span of the organisms with high confidence^76^. Similarly, in humans, lysosomal function and particularly autophagic flux and proteostasis are compromised in T cells with aging^111^. Corroborating the loss of proteostasis with aging, upregulation of proteostasis restorative pathways has shown promising results in aging: an increase in the HSR pathway by upregulating the transcription factor itself increases life span in yeast (chronological aging)^112^ and *C. elegans*^113^. An increase in Mito-UPR in a tissue-specific manner also increases life span^114^. Activating the ERAD arm through IRE1 mitigates the age-dependent collapse of proteostasis^51,115^. This suggests that there is a loss of proteostasis restoration pathways with aging.

If an age-dependent decline in stress-response pathways decreases protein clearance and increases the concentration of damaged proteins, we expect to see a decrease in protein turnover during aging. Autophagic flux in *C. elegans* seems to follow this trend; it decreases with aging and its attenuation works as a mode of rescue in the long-lived mutants^116^. However, protein degradation seems to show a confusing trend: whereas some of the proteins show muted degradation, many others show an increase in degradation, arguing against a general loss of degradation capacity with age^117^. Decreased activity and dysfunction of the proteasome are associated with many late-onset disorders. Furthermore, enhancement in proteasome activity not only extends life span but also increases stress resistance. Thus, a decline in proteasome functionality is an important determinant of aging^98^. The chaperones may be unable to recognize and engage specifically with defective proteins in aged organisms because of post-translational modifications^118–121^. In summary, forced upregulation of quality control branches increases life span. Nevertheless, it remains to be seen whether aging is indeed associated with the muted clearance of defective proteins.

Thus, the role of proteostasis in governing aging and associated phenotypes is still an active field. Given the complex connection between stress-response pathways and the breadth of the response, it is hard to pinpoint a single reason for the age-associated decline in proteostasis and vice versa; most likely, it does not depend on only a single pathway. Because aging depends on multiple pathways, obtaining their quantitative contribution would require quantitative genetic tools that can measure the contribution of each pathway and their epistasis, as has been developed in yeast^122^. This needs to be supplemented with biochemical experiments to allow a holistic understanding of organismal proteostasis and its regulation with aging.

## Role of proteostasis in diseases

### Role of proteostasis in monogenetic diseases

The role of proteostasis is well documented in neurodegenerative diseases^123^, but little is known about its role in monogenetic recessive diseases. Monogenetic recessive diseases linked to protein malfunction are caused by mutations that lead to loss of function of a protein. This can happen if the mutation (1) causes loss of the protein, (2) inactivates the protein by changing its active site, (3) disturbs the normal maturation of the protein, or (4) causes production of a different protein^124^. The majority of the recessive diseases that have been mapped to protein-coding regions do not occur in proteins’ active sites. Although these alterations may still affect protein function by altering allosteric sites or affecting macromolecular interactions^125^, many of them have the potential to affect protein folding and maturation^126,127^. Although its role in the first two cases is not as important, proteostasis may play a major role in the last two cases. HSR activation can cause a maladaptive response primarily for loss-of-function mutations and cause the degradation of mutant proteins^128^. On the same line, some of the disease-causing mutations in Fanconi anemia resulted in mutant proteins that had a stronger association with Hsp70 chaperone machinery than the wild-type (WT) proteins, causing degradation of mutant variants^129^. The work also predicts that mutations that predispose a patient to severe forms of recessive diseases may generally associate more with the Hsp70 chaperone system whereas mild mutations generally tend to associate strongly with the Hsp90 chaperone system^129^. This corroborates nicely with a recent finding which shows that Hsp70 prevents aggregation at the cost of preventing folding of mutant proteins but Hsp90 may relieve this brake and ensure proper maturation^130^. Similarly, cystic fibrosis causing mutations in the channel protein CFTR (cystic fibrosis transmembrane conductance regulator) shows the alleviation of phenotype when the mutant protein is dissociated from its cognate chaperone Aha1^131,132^. However, opposing trends where the upregulation of Hsp70 machinery helped protein maturation have also been observed. Arimoclomol, a proteostasis regulator that amplifies HSR, can decrease the lysosomal dysfunction in cells derived from Gaucher disease–affected patients^133^. Treatment with the drug, as expected, upregulated HSR but interestingly also enhanced the levels of Bip (an ER-resident Hsp70) and helped in the maturation of the mutant GCases (beta-glucosidases) that represented the different mutations found in the patients^133^. So it is difficult to predict the outcome of altering proteostasis on the function of mutant proteins: whereas some mutants may fold more efficiently and regain activity upon induction of proteostasis network members, other mutants may be rendered inactive by the degradation machinery. Nonetheless, proteostasis contributes significantly to the phenotypes of recessive diseases. Large-scale testing with deep mutational scanning of proteins has opened up avenues to understand the role of proteostasis in specific mutations^17,20,134^ and may help in designing tools to efficiently predict the effect of proteostasis modulators on mutants of proteins that cause loss-of-function diseases.

Monogenetic dominant diseases can result from a mutation that either leads to dominant loss of function or forms toxic species. Among the dominant loss-of-function mutations, the most prevalent in cancer is on p53. p53 is stabilized upon DNA damage and ceases cell proliferation^135^. Cells harboring mutations in p53 proliferate and accumulate mutations in the genome. Since p53 forms a tetramer *in vivo*, the mutant proteins are thought to act dominantly by forming oligomers with WT copies of p53. Although many of the p53 mutations have problems binding to DNA muting its transcription factor activity, recent evidence shows that mutant p53 aggregates into amyloid-like fibrils, spreads to neighboring cells, and depletes WT p53 using a prion-like conformational switch mechanism^136^. Thus, if proteostasis alteration modulates protein quality control and aggregation, it is likely to play an important role in the process of p53 amyloidogenesis.

The latter class of monogenetic dominant diseases, that result from a toxic gain of function, includes Huntington’s disease and different classes of spinocerebellar ataxia. These share features of amyloid-like protein aggregation with complex neurological disorders like Alzheimer’s disease (AD) and Parkinson’s disease^123^. For the purpose of this commentary, we are treating both of them as one group comprising diseases showing evidence of amyloid-like protein aggregation. Nevertheless, there is no common pathophysiological background for these diseases; also, different HSPs have been associated with them^137^. For example, each of these diseases has a distinct barcode of HSPs which can rescue the aggregation. Furthermore, these age-related diseases are presented with extracellular or intracellular amyloid aggregates^75^. The aggregated proteins, except for polyglutamine (polyQ)-associated diseases, are divergent in sequence. Generally, repetitive sequences—for example, repeating sequences of amino acids like glycine-alanine dipeptide in amyotrophic lateral sclerosis^138,139^—are more prone to aggregation. Aggregation may also proceed in the absence of any repeat sequences, as in the case of α-synuclein and Aβ. Even for the canonical polyglutamine-containing protein Huntingtin (Htt), recent evidence suggests that toxicity can arise from the regions flanking the polyQ tract^140^ or from repeat-associated non-ATG (RAN) peptides generated by alternate translation frames of CAG repeats^141^. However, the transgenic mice model of Huntington’s disease exhibited toxicity only due to the aggregation of polyQ peptides and not due to RAN translated repeats^142^. Thus, identifying the toxic species in aggregation-associated diseases is still an active field. The target organs with different mutant proteins are different. For example, mutant Htt with polyQ extension in exon 1 aggregates in the cortex and striatum region of the brain while islet amyloid polypeptide (IAPP), an aggregate associated with type II diabetes, is found in the pancreas. For many of these diseases, the mutant protein is ubiquitously expressed in different regions of the body but the puzzling feature is the specificity of the site of aggregation. Although the late-onset nature of aggregation seems to be explained by the failing proteostasis, there is no consensus on the spatial specificity of aggregation^72^.

Many amyloid aggregates that are naturally found are not toxic^143^. Then why do some mutant protein aggregates show pathology associated with them? One possibility is that the aggregated proteins are not toxic but the soluble misfolded proteins, in either monomeric or oligomeric form, are toxic for the cells^144^. Misfolded proteins may sequester chaperones and make them unavailable for other essential substrates^27,145^. Mutant proteins like Htt form toxic soluble oligomers and seed further aggregation^146–148^. Thus, soluble oligomers extending to form large intracellular protein aggregates seem to be a recurrent feature of many late-onset aggregation-prone diseases. Although soluble aggregates play a major role in toxicity, both insoluble and soluble aggregates are toxic for the cells *ex vivo* in many of the canonical diseases^75^. Aggregates can also cause toxicity by permeabilizing membranes^149^ or sequestering other macromolecules^150^. Recent evidence also suggests that a large-scale metabolism change due to toxic misfolding in cells results from mitochondrial dysfunction^151^. Thus, toxicity seems to be multipronged and may depend partially on the aggregating protein, the spatial location of aggregates, and environmental stimuli.

Why do these toxic species accumulate? This may happen if these species are either overexpressed or hoarded by blocking the clearance pathway. Many of these aggregates are known to inhibit proteasomal activity, thereby inhibiting their clearance. For example, soluble aggregates of different pathological aggregation-prone proteins have similar structural features that enable them to target proteasome and block their activity with high affinity^152^. Some mutant proteins, like toxic oligomers of mutant Htt that bypass chaperone-mediated autophagy, may avoid clearance^153^. PolyQ expanded proteins may also block autophagy by decreasing Beclin-1 that is essential for autophagy. Beclin-1 (a key regulator of autophagy) interacts with the polyQ tracts of Ataxin-3 that prevents its degradation^154^. Expression of other proteins with polyQ tracts compete this interaction in a polyQ length-dependent manner, thereby causing Beclin-1 to be degraded by the proteasome. This leads to a decline in autophagic flux in cells harboring polyQ tracts in mice as well as human brain cells^154^. Proteotoxicity is resolved when autophagy block is relieved artificially, suggesting that the clearance arms of proteostasis are key to prevent the accumulation of these toxic proteins. As a corollary, conditions that compromise clearance mechanisms should precipitate the disease. Indeed, viral infections that block autophagy have been shown to precipitate α-synuclein aggregation^155^. However, the interdependence between autophagy and proteasomal degradation and between different branches of the autophagy pathways warrants more careful studies in unraveling the role of specific branches in the clearance of misfolded proteins, diffusely aggregated protein, insoluble protein aggregates, or organelles burdened terminally with proteotoxicity^156–158^.

Protein misfolding is known to induce an appropriate response by increasing the clearance capacity of the cells. Then why does it fail in these diseases? Failing response with age, as discussed above, could be the clue making these diseases late-onset diseases. However, these oligomers themselves have been found to perturb the proteostasis network and blunt cellular response, leading to a faster collapse of proteostasis with aging. Aggregates of α-synuclein found in patients with Parkinson’s disease can facilitate degradation of HSF1, thereby diminishing the sensory mechanisms that can mount a defensive response^159,160^. Aggregates of mutant Htt interact with the TIM23 translocase of mitochondria, thus perturbing mitochondrial proteostasis and possibly preventing its degradation through the MAGIC pathway^45^. Perturbed mitochondrial homeostasis may be a common crucial mediator in these diseases as even in sporadic cases of AD (without mutations in the known modulators), mito-UPR genes were found to be upregulated, attesting to the general role of mitochondrial proteostasis in AD^161^. These studies provide us with valuable knowledge of the underlying problems in these diseases, which have set forth new avenues that might lead to therapy.

### Role of proteostasis in infectious diseases

Many intracellular pathogens use host cellular machinery for the efficient maturation of their proteins. This is particularly true for viruses that use the host chaperones to create their proteostasis network that aids viral replication. For example, ER-resident chaperones are important for the efficient maturation of viral surface proteins, and cytosolic chaperones are important for the folding of many of the non-structural proteins^162,163^. Large polyproteins made by viruses need to be processed and folded correctly. Mounting evidence shows that the maturation of large proteins is dependent upon the host chaperones like Hsp90^162,164^. The autophagy pathway is also crucial for generating replication sites for different viruses^165^. Interestingly, some of the viral protein aggregates are known to increase autophagy in the infected cells whereas others block the process of autophagy. Given the importance of the proteostasis machinery of the host in viral replication, the ER proteostasis inhibitor Castanospermine has shown pan-antiviral activity against many enveloped viruses^166^, and Hsp70 inhibitor has been shown to have strong anti-viral activity against the Zika virus^163^. Drug resistance in many viruses emerges fast as they can accumulate mutations rapidly. Many of the coding missense mutations can likely compromise protein folding, causing their evolution dependent upon the host proteostasis machinery. It has been shown that the mutations accumulated on the Influenza virus are regulated by the host proteostasis^134^ and inhibiting proteostasis prevents the emergence of drug-resistant Zika viruses^163^. Given the different networks present in different compartments, compartment-specific host proteostasis can show different effects on viral evolution^167^. Interestingly, a mutation (Pro283 nucleoprotein variant) fixed in the human Influenza virus strain, which provided immune tolerance, was found to be inactivated upon inhibition of HSF1 at febrile temperatures^168^. This proved that the proteostasis network may play an important role in buffering mildly deleterious mutations that have some functional advantage. However, HSF1, as mentioned earlier, controls many pathways, including the chaperone network. Thus, it remains to be seen whether this mutant takes the help of chaperones to fold *in vivo*. Intracellular bacteria communicate with the host cells through their secretory proteome. Deubiquitninases (DUBs) are among the prominent classes of enzymes secreted by bacteria that rewires the host proteostasis network to deubiquitinate proteins preventing the growth of intracellular pathogens^169^.

**Figure 4.**
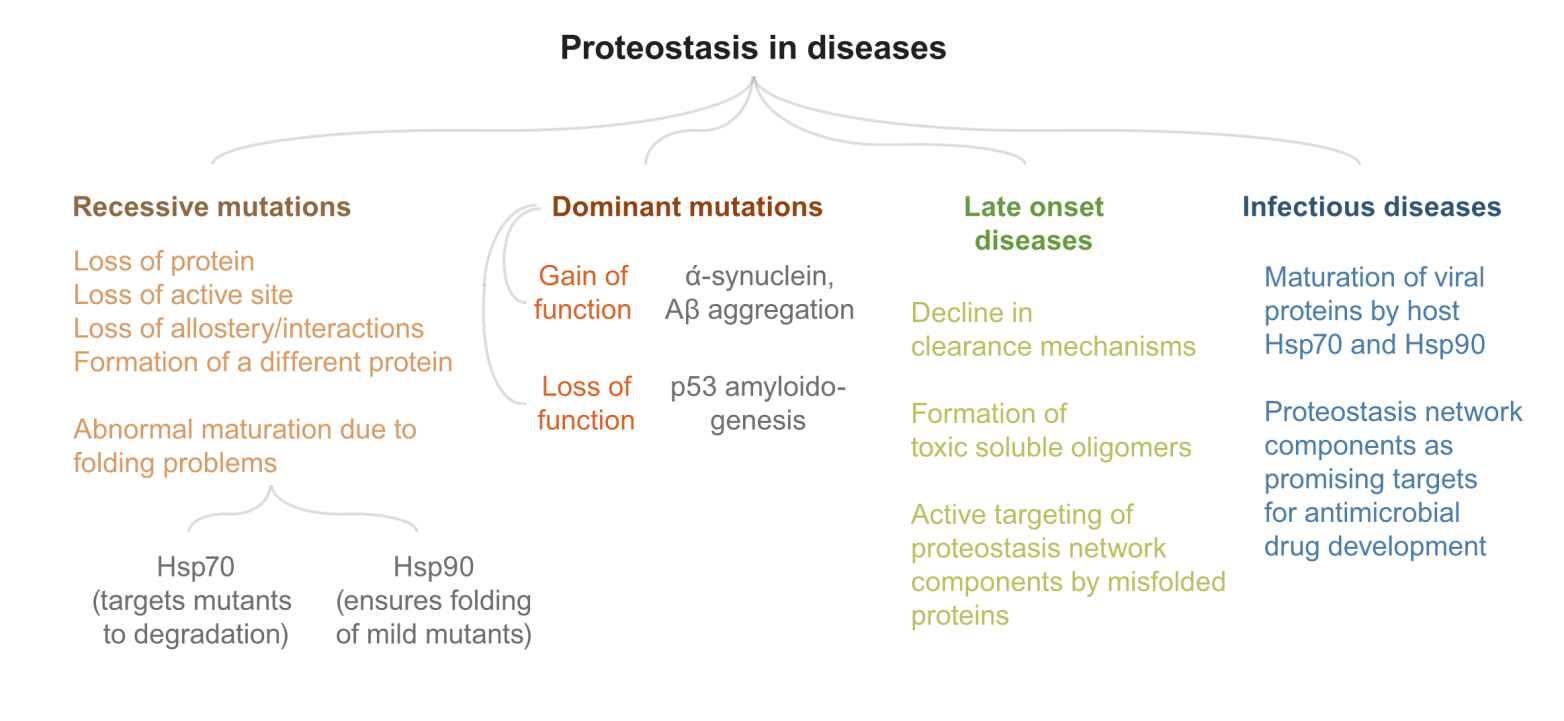
Proteostasis in diseases. A summary of proteostasis-related diseases is presented. Many of the proteostasis-related diseases we know of have a genetic component. There has been considerable progress in deciphering the molecular mechanisms of many of these diseases. Still, a better understanding of eukaryotic stress-response pathways will take us a long way in the direction of treatment and cure.

For extracellular pathogens like bacteria, proteostasis network members are major targets for the development of anti-microbial drugs. CLPP activators that constitutively activate CLPP protease and cause proteostasis imbalance are interesting candidates for antibiotics^170^. Bacteria can tolerate anti-microbials and evolve anti-microbial resistance (AMR). AMR can be affected by bacterial dormancy, which in turn results in persistence. In an intriguing study, Fan Bai’s group showed that protein aggregates determine bacterial dormancy and stress tolerance linking the role of proteostasis to stress-based cellular memory and possibly AMR^171^. Given that proteostasis members, chaperones (protein and chemical), can buffer mutations, it is tempting to speculate that alteration in the concentration of these members assists in the evolution of AMR. Intriguingly, metabolic alterations associated with osmotic shock have been shown to alter mutational buffering^17^. Thus, changes in metabolism either due to change in the niche of a microbe or due to the metabolic rewiring can potentiate genetic variations that may culminate in AMR. A summary of the above-mentioned diseases is shown in Figure 4.

## Outlook

Long-term quests in the field have been to understand the role of proteostasis in regulating different physiological or pathological processes and to identify proteostasis-modulating strategies to alleviate problems associated with these processes. This would require the painstaking effort to catalogue the dependence of the different pathways on proteostasis. A specific problem that requires attention is the way that proteostasis is perturbed to investigate the dependence. Genetic deletion of chaperones or other members of the proteostasis network is often used to investigate the dependence, but their deletion most often upregulates other pathways of proteostasis that do not optimally complement the loss of the deleted pathway but rather increase the clearance processes^172^. The results obtained upon chaperone deletions in *S. cerevisiae* are hence convoluted; they could be an outcome of the loss of the pathway or a dominant effect of the upregulated pathways. So it is important to devise strategies to effectively downregulate functions of chaperones without complete deletion, a strategy that has already produced interesting results^173^.

To identify novel proteostasis modulators, we need to have comprehensive knowledge of pathways regulating proteostasis. There is a large gap of knowledge in our understanding of the pathways that regulate proteostasis in higher eukaryotic systems. For example, although we know that the deletion of some of these pathways (like certain branches of ER-UPR) can cause specific effects in certain tissues, we do not understand the reason that these pathways show tissue specificity although these are expressed ubiquitously in different tissues^174,175^. We do not know whether there is a threshold load of misfolded proteins that activate the different branches differentially. It is also possible that each of these pathways has a tissue-specific difference in their threshold of activation, a phenomenon that can change the way we target the pathways in different diseases. In fact, we understand little about how the response to proteostasis perturbations is guided in a cell type–specific manner.

However, the cumulative knowledge generated in this field has allowed the development of multiple therapeutic candidates that can be used to ameliorate human suffering^176–179^. As we unravel the role of proteostasis in different biological settings and close the gaps in knowledge, we hope that the excitement of discovery will be surpassed only by the usefulness of the discoveries in making our planet far more livable.
